# Molecular Cloning and Expression Analysis of *fushi tarazu* Factor 1 in the Brain of Air-Breathing Catfish, *Clarias gariepinus*

**DOI:** 10.1371/journal.pone.0028867

**Published:** 2011-12-28

**Authors:** Parikipandla Sridevi, Aparna Dutta-Gupta, Balasubramanian Senthilkumaran

**Affiliations:** Department of Animal Sciences, School of Life Sciences-Centre for Advanced Studies, University of Hyderabad, Hyderabad, India; University of Hyderabad, India

## Abstract

**Background:**

*Fushi tarazu* factor 1 (FTZ-F1) encodes an orphan nuclear receptor belonging to the nuclear receptor family 5A (NR5A) which includes adrenal 4-binding protein or steroidogenic factor-1 (Ad4BP/SF-1) and liver receptor homologue 1 (LRH-1) and plays a pivotal role in the regulation of aromatases.

**Methodology/Principal Findings:**

Present study was aimed to understand the importance of FTZ-F1 in relation to brain aromatase (cyp19a1b) during development, recrudescence and after human chorionic gonadotropin (hCG) induction. Initially, we cloned *FTZ-F1* from the brain of air-breathing catfish, *Clarias gariepinus* through degenerate primer RT-PCR and RACE. Its sequence analysis revealed high homology with other *NR5A1* group members *Ad4BP/SF-1* and *LRH-1*, and also analogous to the spatial expression pattern of the latter. In order to draw functional correlation of *cyp19a1b* and *FTZ-F1*, we analyzed the expression pattern of the latter in brain during gonadal ontogeny, which revealed early expression during gonadal differentiation. The tissue distribution both at transcript and protein levels revealed its prominent expression in brain along with liver, kidney and testis. The expression pattern of brain *FTZ-F1* during reproductive cycle and after hCG induction, *in vivo* was analogous to that of *cyp19a1b* shown in our earlier study indicating its involvement in recrudescence.

**Conclusions/Significance:**

Based on our previous results on *cyp19a1b* and the present data, it is plausible to implicate potential roles for brain *FTZ-F1* in ovarian differentiation and recrudescence process probably through regulation of *cyp19a1b* in teleosts. Nevertheless, these interactions would require primary coordinated response from ovarian aromatase and its related transcription factors.

## Introduction

*Fushi tarazu* factor 1 (FTZ-F1) is an orphan nuclear receptor [1], [2] that was initially identified as an activator of *fushi tarazu*, a pair-ruled homeobox gene involved in the segmentation of *Drosophila* [2]. Since then numerous *FTZ-F1* homologues have been recognized in several species. Presently, FTZ-F1 constitutes a distinct subfamily, NR5A, in the superfamily of nuclear receptors [3]. It includes two major subgroups of related genes with separate function and expression patterns in higher vertebrates. The NR5A1 subgroup contains the FTZ-F1 homologue, which also include adrenal 4-binding protein (Ad4BP) [4] or steroidogenic factor-1 (SF-1) or Ad4BP/SF-1 [5]. In vertebrates, biosynthesis of steroid hormones is regulated by the tissue-specific expression of cytochrome P450 (CYP) enzymes [6] and analysis of the promoter motifs of *CYP* and steroid hydroxylase genes revealed the presence of elements specific for a transcription regulator, FTZ-F1 [7], [8]. Ad4BP/SF-1, a homologue of FTZ-F1, has been identified as an important regulator of steroidogenesis due to its regulatory influence on several CYP enzymes involved in steroidogenic pathways [9]. Hence, this group of transcription factors is critical for normal physiological entrainment of hypothalamo-hypophyseal-gonadal axis during reproduction and sexual differentiation in vertebrates [10]. The NR5A2 subgroup contains the liver receptor homologue 1 (LRH-1) or α-feto protein transcription factor (FTF), which regulates the expression of the α-feto protein [11] and is involved in cholesterol metabolism of mammals [12].

In non-mammalian vertebrates, role of *FTZ-F1* has not been fully understood. Teleost FTZ-F1 has been put forward as a likely candidate for upstream regulation of several genes involved in steroidogenesis, and it has been suggested that teleost *FTZ-F1* may be involved in feminization of developing testis in response to estrogen exposure [13]. The expression patterns of teleost *FTZ-F1* homologues partly correlate with mammals [14], [15], but their role in reproduction is yet to be elucidated in detail, although few reports are available in teleosts [16], [17], [18].

In several teleosts, FTZ-F1 and its homologues are reported to be involved in the regulation of either form of aromatases [4], [5], [18], [19], [20]. Expression pattern of *FTZ-F1* has been reported in medaka [18] and zebrafish [21], yet studies regarding its protein expression are limited and that too in brain. This prompted us to clone *FTZ-F1* from the brain of air-breathing catfish, *Clarias gariepinus*, which is an annual breeder. Catfish are excellent teleost model due to its seasonal gonadal attenuation and recrudescence, which mimics recurrence of sexual maturation. Thus, the events of gonadal ontogeny and gonadal cycle can be compared explicitly unlike fishes which retain maturity throughout life cycle or undergo rapid changes of gonadal cycle. Brain-pituitary-gonadal axis is well coordinated like many other seasonally breeding teleosts. We further analyzed *FTZ-F1* expression pattern in brain during reproductive cycle and after human chorionic gonadotropin (hCG) induction, *in vivo* to correlate the events of ovarian cycle. Western blot analysis was performed to analyze changes at protein level using purified FTZ-F1 antibody raised against an antigenic peptide designed from deduced amino acid sequence of catfish *FTZ-F1*. Our observation of specific expression of *FTZ-F1* in brain but not in ovary like that of brain aromatase (*cyp19a1b*) in catfish [22], opens up new avenue of research to unravel the regulatory role of the former over the latter.

## Results

### Cloning of *FTZ-F1* from brain of catfish

**Figure 1:**
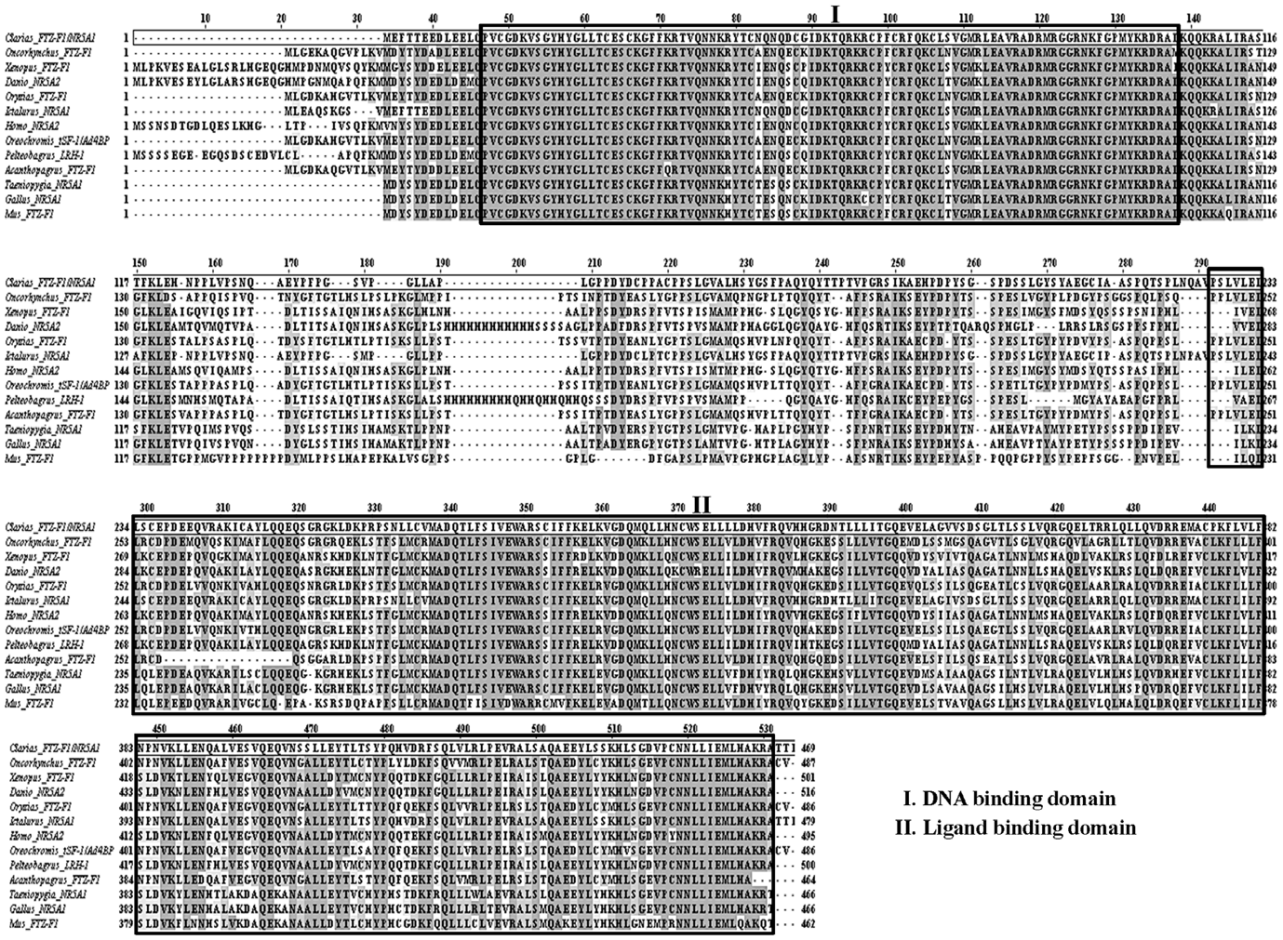
ClustalW alignment of deduced amino acid sequence of catfish FTZ-F1 with other vertebrate counter parts. The alignment was done using software ClustalW (EBI tools). Shaded region represents conserved amino acids and signature domains are represented by rectangle boxes. GenBank accession numbers of the sequences we used are as follows: *Clarias gariepinus*; JN859075, *Ictalurus punctatus*; DQ000612, *Oreochromis niloticus*; AB060814, *Oryzias latipes*; AB016834, *Oncorhynchus mykiss*; NM_001124537, *Danio rerio*; NM_131463, *Acanthopagrus schlegeli*; AY491379, *Taeniopygia guttata*; NM_001076692, *Pelteobagrus fulvidraco*; EU860284, *Xenopus laevis*; BC169770, *Gallus gallus*; NM_205077, *Mus musculus*; AF511594 and *Homo sapiens*; BC118571.

**Figure 2:**
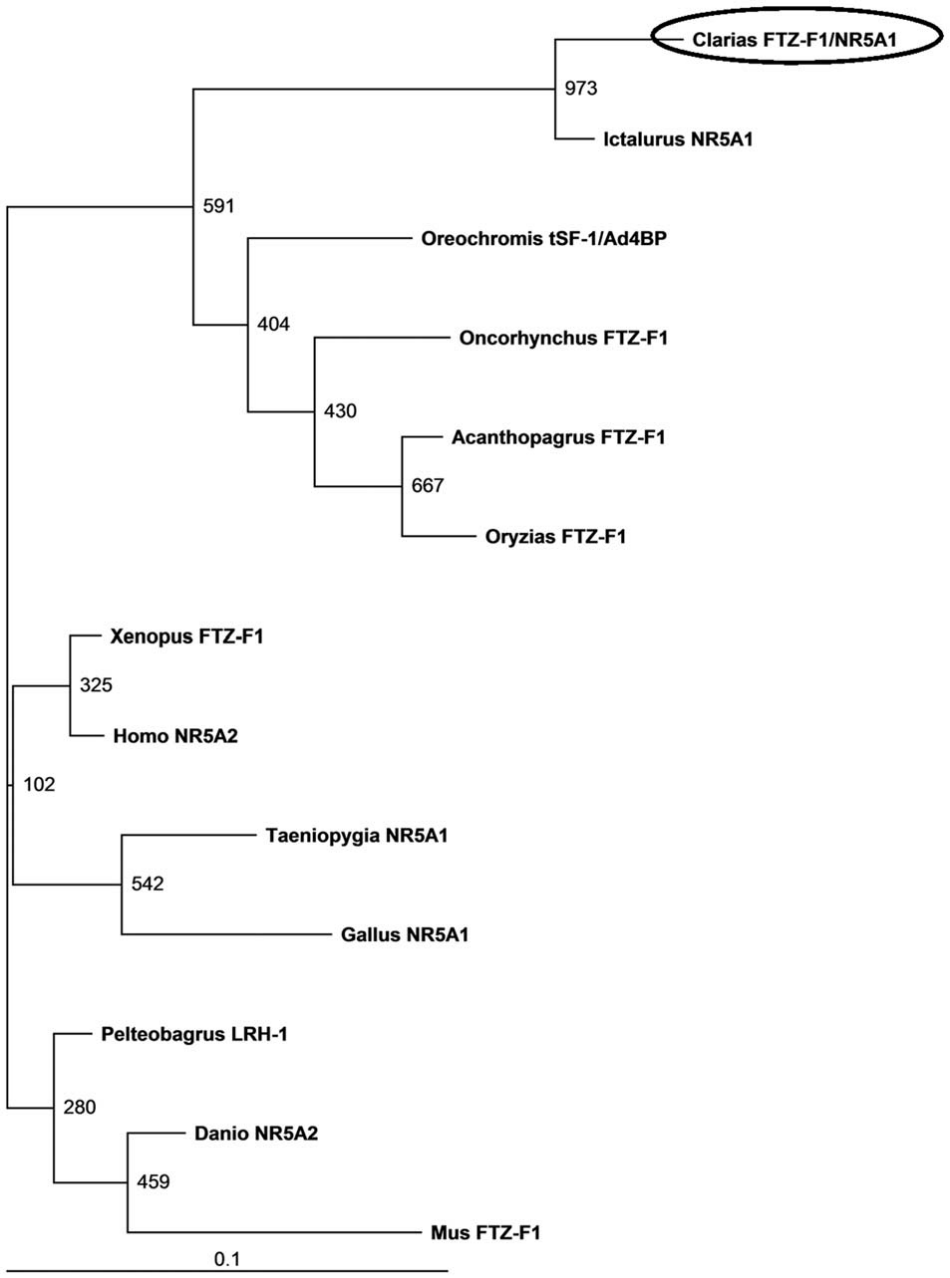
Phylogenetic tree showing the evolutionary status of catfish FTZ-F1. Phylogenetic tree constructed using NJ method and bootstrap analysis with 1000 replicates was used to assess the strength of nodes in the tree. Phylogenetic analysis was done using ClustalW software of DNA data bank of Japan and supported by Tree view software. The scale bar represents 0.1 substitutions per amino acid site. GenBank accession numbers of the sequences used in the analysis are indicated in Fig. 1.

A partial cDNA of 243 bp was obtained using a set of degenerate primers (DeFw and DeRv, Table 1). The sequence identity of the fragment was confirmed by NCBI-BLAST. Full-length *FTZ-F1* cDNA obtained by using 5′ and 3′ RACE (Refer Table 1 for primers) was 2355 bp long with 319 bp of 5′ untranslated region (UTR), 626 bp 3′ UTR and polyadenylation signal ATTAAA which is 24 bp upstream of poly-A tail. The open reading frame (ORF) is 1410 bp long, encoding 469 amino acid residues. Nucleotide sequence of *FTZ-F1* was deposited in the GenBank under the accession no. JN859075. ClustalW alignment revealed the presence of DNA binding, ligand-binding/dimerization and transactivation (AF-2) domains, which are conserved in the transcription factors belonging to nuclear receptor superfamily, are well preserved in catfish (Fig. 1). Phylogenetic analysis revealed that catfish FTZ-F1 is evolutionarily closer to the counterparts of channel catfish, *Ictalurus punctatus* and the Nile tilapia, *Oreochromis niloticus* (Ad4BP/SF-1), and showed high homology with *NR5A1* genes of most teleosts and other vertebrates (Fig. 2).

**Table 1:** List of primers used for cloning and expression analysis of catfish brain *FTZ-F1*.

Sl. No.	Primer name	Nucleotide sequence 5′ to 3′	Purpose
1.	De Fw	GCWCTVCGTGATKGCRGACYAGASKCTG	To amplify partial cDNA fragment using degenerate primers
2.	De Rv	GAKAGYCCAGWCCCGASTCAGAMAYGMCG	To amplify partial cDNA fragment using degenerate primers
3.	5′ P	CTGATCTCCGACCTTTAGCTCC	5′ RACE
4.	5′ N	GTCTGGTCCGCCATCACGCAG	5′ RACE
5.	3′ P	GACAGGTGCATCATGGAAGAGAC	3′ RACE
6.	3′ N	GAGGTTGAGCTCGCGGGCGTC	3′ RACE
7.	RT-Fw	GGAGGAGCTCTGTCCTGTGTGCG	qRT-PCR
8.	RT-Rv	GCTGCGTCTTATCGATGCCGCAG	qRT-PCR
9.	â-actin Fw	ACCGAATGCCATCACAATACCAGT	qRT-PCR
10.	â-actin Rv	GAGCTGCGTGTTGCCCCTGAG	qRT-PCR

***Note:*** The abbreviations (underlined) for the degenerate bases used in primers, 1 and 2 are **W**  =  A or T; **V**  =  A, C, or G; **R**  =  A or G; **S**  =  G or C; **K**  =  G or T; **Y**  =  C or T; **M**  =  A or C.

***Other abbreviations:*** **De** =  Degenerate; **Fw** =  Forward; **Rv** =  Reverse; **P** = Primary; **N** = Nested; **RT** & **qRT-PCR** = Real time PCR; **RACE** =  Rapid Amplification of cDNA Ends.

### Western blot analysis

**Figure 3:**
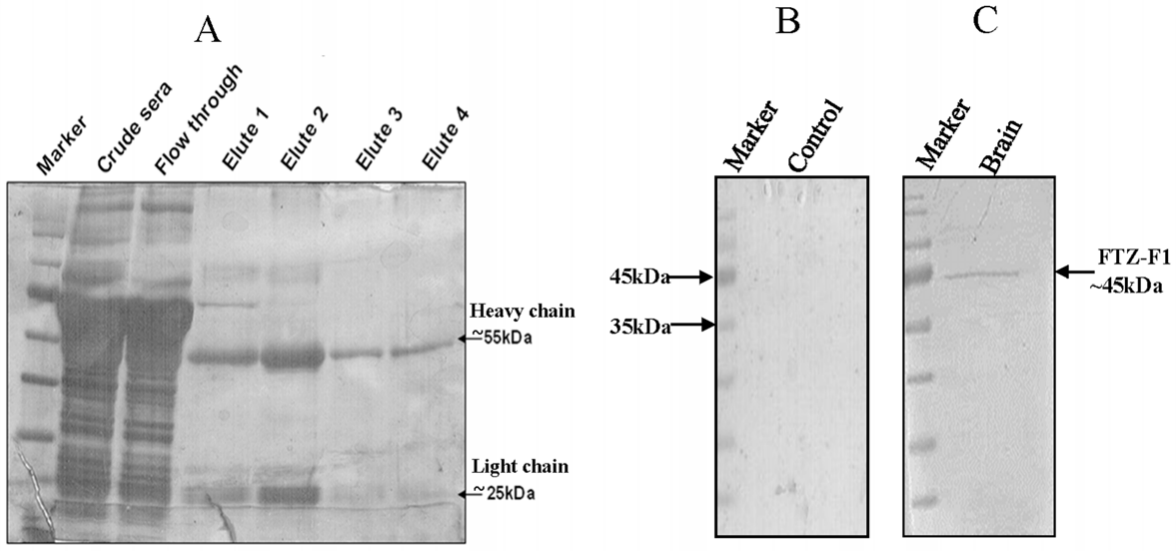
Peptide affinity purification profile of FTZ-F1 antibody and Western blot analysis. A) IgG fraction of FTZ-F1 antibody showing band at ∼55 kDa (heavy chain) and ∼25 kDa (light chain). B) No signal was seen in negative control. C) A protein band at ∼45 kDa corresponding to deduced FTZ-F1 protein detected in the female brain protein homogenate of preparatory phase catfish.

The purification profile of FTZ-F1 antibody (IgG fraction) heavy and light chains is shown in Fig. 3A. Synthetic peptide pre-incubated with the purified fraction of antibody was used as a negative control to determine its specificity against FTZ-F1 protein (Fig. 3B). The antibody detected a ∼45 kDa protein from the segregated gel purification of catfish brain protein homogenate, which matched with predicted size of FTZ-F1 protein of catfish (Fig. 3C).

### Tissue distribution pattern of *FTZ-F1*

**Figure 4:**
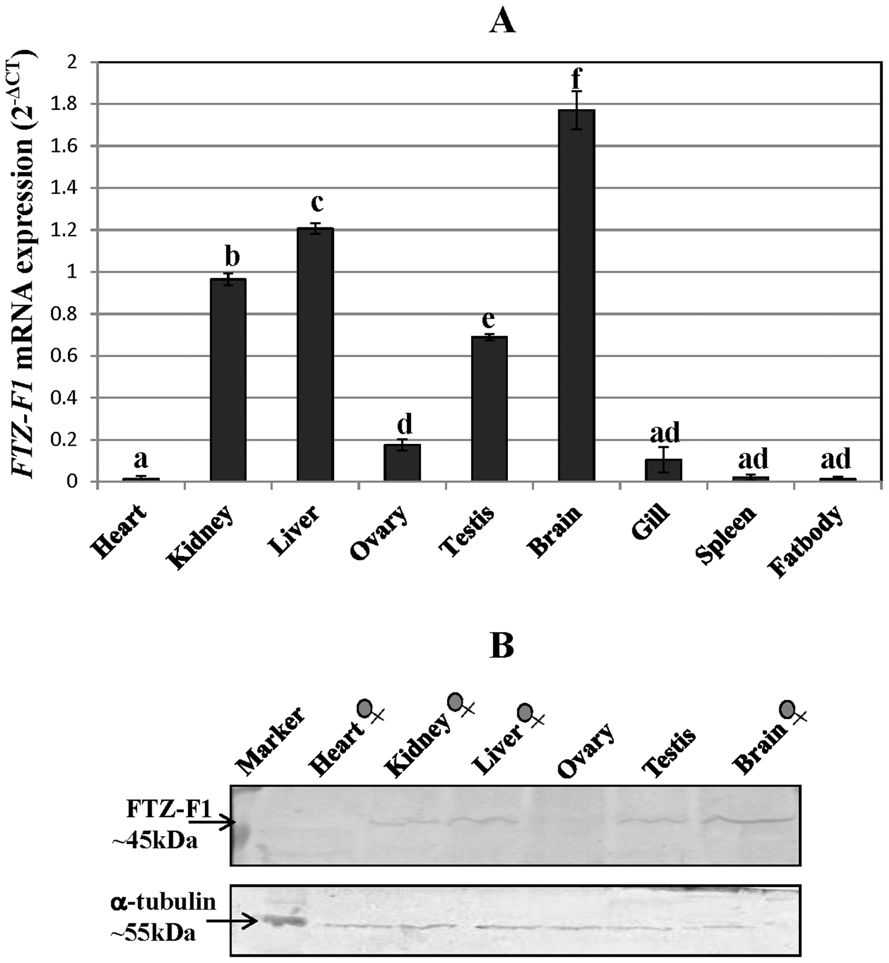
Tissue distribution of *FTZ-F1* in adult female catfish. A) qRT-PCR analysis of *FTZ-F1* expression (reported as 2^−ΔCT^) was done in different tissues of prespawning phase female catfish along with testis of prespawning phase male catfish. Values are mean ± SEM, n = 5. Means with different letters differ significantly and are compared group-wise (*P*<0.05). B) Tissue distribution pattern of FTZ-F1 by Western blot. As explained above, different tissues of female catfish and testis with FTZ-F1 antibody in upper lane, α-tubulin antibody in lower lane as control to show equal loading.

The tissue distribution pattern of *FTZ-F1* was analyzed by qRT-PCR showed highest expression in brain (Fig. 4A). Low levels of expression were also seen in liver, kidney and testis, while it was negligible in ovary and gills. The tissue distribution pattern of FTZ-F1 protein correlated well with that of the transcript levels when probed with the FTZ-F1 antibody (Fig. 4B).

### Ontogeny of *FTZ-F1* in catfish brain

**Figure 5:**
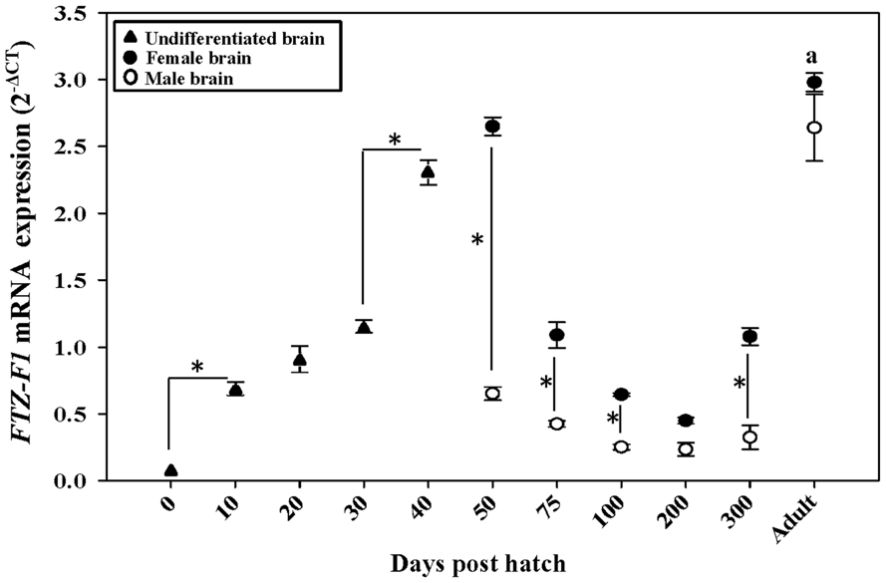
Catfish brain *FTZ-F1* expression during gonadal ontogeny by qRT-PCR. Quantitative analysis of brain *FTZ-F1* expression relative to *β-actin* expression during gonadal ontogeny of male and female catfish is reported as 2^−ΔCT^. Values are mean ± SEM, n = 5. *indicates the significance at *P*<0.05, **^a^**denotes the significance at *P*<0.05 from 50/75 - 300 dph to adult for respective sex.

The expression of *FTZ-F1* was monitored from day 0 (less than 24 h post hatch) to adult stage at different time intervals in brain by qRT-PCR (Fig. 5) which revealed dimorphic pattern between sexes. In female brain, the expression increased from 0 to 40 days post hatch (dph) and peaked to maximum by 50 dph. Thereafter, expression of brain *FTZ-F1* gradually decreased from 50 to 200 dph, and elevated again at 300 dph and adult stage. In the case of males, expression decreased drastically from 50 to 300 dph and elevated further in the adult stage. The expression pattern of *FTZ-F1* was higher in adult brain when compared to juvenile brain, sex-wise except for 50 dph female brain (Fig. 5).

### Expression of *FTZ-F1* during reproductive cycle

**Figure 6:**
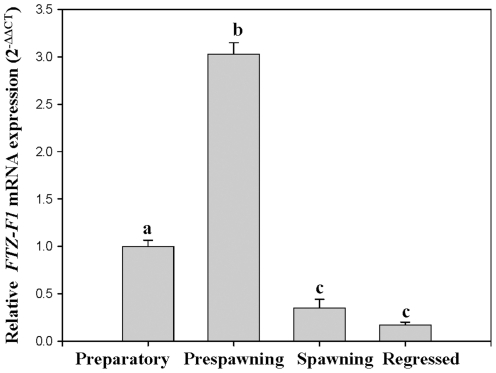
Expression of *FTZ-F1* in brain during different phases of catfish ovarian cycle. Quantitative analysis of brain *FTZ-F1* expression relative to *β-actin* expression during different phases of ovarian cycle was reported as fold change relative to preparatory phase calculated using 2^−ΔΔCT^ method. Values are mean ± SEM, n = 5. Means with different letters differ significantly and are compared group-wise (*P*<0.05).

*FTZ-F1* expression was analyzed in brain using qRT-PCR (Refer Table 1 for primers) during different phases of reproductive (ovarian) cycle of female catfish (Fig. 6). Expression of *FTZ-F1* was highest during prespawning phase. The expression was found to be lowest in the regressed phase and moderate in other phases.

### Expression of *FTZ-F1* after hCG induction, *in vivo*

**Figure 7:**
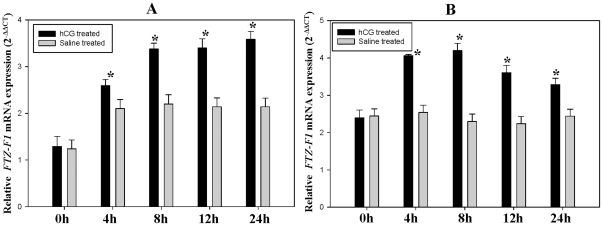
Expression of *FTZ-F1* in catfish brain at different time points after hCG induction, *in vivo*. Quantitative analysis of brain *FTZ-F1* expression relative to *β-actin* expression in A) Preparatory and B) Prespawning phases of ovarian cycle compared to saline treated controls by qRT-PCR was reported as fold change relative to 0 h calculated using 2^−ΔΔCT^. X-axis represents hours after treatment. Values are mean ± SEM, n = 5. * indicates significance at *P*<0.05.

Brains collected at 0, 4, 8, 12 and 24 h after hCG induction, *in vivo* showed a gradual increase in the level of *FTZ-F1* transcripts from 0 to 8 h, that was persistent till 12 h during the preparatory phase (Fig. 7A). In the prespawning phase, the expression was found to be highest at 4 h and decreased gradually at later time points when compared to saline treated controls (Fig. 7B).

## Discussion

ClustalW alignment of the deduced amino acid sequence of cloned catfish FTZ-F1 with its vertebrate counterparts indicated the presence of typical signature domains such as DNA-binding, ligand-binding/dimerization and AF-2 of nuclear receptor family including NR5A [4], [23]. Despite numerous studies related to *FTZ-F1* genes in the past in silkworm [1], [24], zebrafish [15], [21], rainbow trout [25], the African clawed frog [26], chicken [27], mouse [8], rat [10], [28], bovine species [4], human [29] and shrimp [30], there exists a discrepancy in the nomenclature and function of these genes. Multiple forms of *FTZ-F1* have been reported across vertebrate phyla that includes four isoforms in zebrafish [15], [21], [31] and two isoforms in the arctic char [17], [32], frog [33] and chicken [27]. In the present study, only a single form of *FTZ-F1* was obtained similar to rainbow trout [25], medaka [18] and orange spotted grouper [34]. The deduced amino acid sequence and phylogenetic analysis of catfish *FTZ-F1* showed identity with *NR5A1* genes. Hence, we designated this clone as *FTZ-F1/NR5A1*.

The tissue distribution analysis of *FTZ-F1* revealed its expression predominantly in brain with low levels in liver and kidney, specifying its pattern similar to *LRH-1* and *FTZ-F1* in other teleosts [19], [32]. Expression in catfish testis analogous to that of black porgy and zebrafish signifies its role similar to that of *Ad4BP/SF-1* in male teleosts [15], [35]. Further, similar expression pattern of *FTZ-F1* was also reported in zebrafish where zFF1a was expressed in different brain regions [15] and zFF1b in pancreas and diencephalon region of brain [36]. These were also analogous to *FTZ-F1* expression in orange spotted grouper where it was found in forebrain, hypothalamus and pituitary, whose function is similar to *SF-1* in mammals [37]. Negligible *FTZ-F1* expression in ovary warrants for the presence of other isoforms like *Ad4BP/SF-1* in that tissue as ovarian aromatase (cyp19a1a) is primarily regulated by this correlate in the Nile tilapia [5]. Further studies are needed to confirm this contention in catfish.

Ontogeny of FTZ-F1 has been studied in various tissues including brain of teleosts [15], [32], [35], [36], although the function and expression patterns are diverse. In this study, we observed an early expression of *FTZ-F1* in brain which reached a maximum at 50 dph which corroborates with late critical window period (35-50 dph) of sex differentiation in catfish [38]. The other highlight was the dimorphic expression pattern of *FTZ-F1* similar to that of *cyp19a1b* [22]. Previous reports in medaka, rainbow trout and mice predicted a role for FTZ-F1 and its homologues, SF-1 and LRH-1, in sex differentiation and reproduction [9], [19], [25], [37]. A role for cyp19a1b in ovarian differentiation and recrudescence through brain-pituitary-gonadal axis has been suggested in catfish [22]. Interestingly, the expression pattern of brain *FTZ-F1* in the present study correlates with that of *cyp19a1b* [22] during reproductive cycle, ovarian development and also after *in vivo* hCG induction. Treatment of fish with luteinizing hormone (LH) or its analogues such as hCG in the preparatory and prespawning phases, leads to the promotion of vitellogenesis [5], [39]. This was not observed during spawning phase due to the shift in steroidogenesis in full-grown immature oocytes to undergo meiotic maturation [5], [40], [41], [42]. Hence, the regulation of estrogen production by gonadotropins with shift in steroidogenesis is considered important from vitellogenesis to maturation [5], [40], [41], [42]. In this regard, the primary target of LH is *cyp19a1a* [5], [22], [39] in ovary which might indirectly influence *cyp19a1b* in brain [22] as it is the rate-limiting enzyme for estrogen biosynthesis. More recently, we found a strong correlation between *cyp19a1b* and *FTZ-F1* expression during brain development and also reported that FTZ-F1 up-regulates the *cyp19a1b* transcription by specific binding to the promoter motifs [43]. Taken together, FTZ-F1 might play crucial roles in ovarian differentiation, development and recrudescence via brain-pituitary-gonadal axis in cooperation with cyp19a1b. Nevertheless, cyp19a1a and FOXL2 in ovary would be primarily responsible for ovarian differentiation, development and recrudescence [22], [43], [44].

In summary, we cloned *FTZ-F1* from the brain of catfish. Based on the deduced amino acid sequence homology and structural features, it can be considered as a potential nuclear receptor belonging to the NR5A1 subfamily. Our results on expression pattern of *FTZ-F1* in brain during gonadal ontogeny and reproductive cycle corroborates well with *cyp19a1b* expression in brain [22], and *cyp19a1a* [22] and *FOXL2* [44] expression in ovary of catfish. Taken together, our results indicate potential roles for *FTZ-F1* in ovarian differentiation and recrudescence probably through the regulation of *cyp19a1b* yet primary coordinated responses from *cyp19a1a* and its related transcription factors are indispensable.

## Methods

### Animals and sampling

Various age groups of *C. gariepinus* were obtained from our laboratory aquaculture facility. Catfish breeding, rearing and sample collection procedures were described earlier [38], [44]. In brief, catfish were kept in glass water tanks supplied with filtered water and maintained under ambient photothermal conditions. They were fed tubeworms, *ad libitum* till they became fingerlings (5–6 mm). They were then provided minced goat liver or pelleted fish food till adulthood (around 400 dph). The catfish hatchlings were sacrificed at different time points i.e., 0 (less than 24 h post hatch), 5, 10, 20, 30, 50, 75, 100, 200 and 300 dph, and adult by briefly immobilizing them in mild ice-cold water dissolved with MS222 (Sigma, St. Louis, MO, USA). Brain was dissected out using fine scissors and forceps under stereo zoom microscope (Leica, Germany) except at day 0 wherein whole body was used. Since it was not possible to isolate brain from 10 and 20 dph larvae, we used entire head for total RNA preparation. All catfish experiments and sacrifice procedures were done by following the general animal ethical guidelines of Institutional Animal Ethical Committee (IAEC), however approval is not required for edible fish sacrifice. Five biological samples (n = 5) obtained from pooled brain tissues of 3-4 larvae for each sample were used for each time point of ontogeny study. To perform seasonal cycle studies, adult fishes (n = 5) were collected in the months of February, May, September and December corresponding to preparatory, prespawning, spawning and regressed phases, respectively to dissect out brain as explained above. All the tissue samples collected were snap frozen in liquid N_2_ and stored briefly at −80°C until use.

### Molecular cloning of *FTZ-F1* from catfish brain

Total RNA from brain/other tissues of juvenile/adult catfish was isolated using TRI-reagent (Sigma). The quality and concentration of total RNA was assessed by using a NanoDrop (ND-1000, NanoDrop technologies, USA) spectrophotometer and checked in formaldehyde agarose gel. Total RNA from adult tissue was reverse transcribed to obtain first strand cDNA using Superscript III (Invitrogen, Carlsbad, CA, USA) and oligodT_18_ primers following the manufacturer's instructions. The efficiency of the transcription was checked by performing a PCR for *β-actin,* a constitutively expressed gene. Based on the alignment of known *FTZ-F1* sequences using Lasergene software (release 3.05; DNASTAR, Madison, WI, USA), a set of degenerate primers were designed and synthesized. Using degenerate primers (De Fw and De Rv primers, Table 1) a partial cDNA fragment was amplified from brain. The amplicon was then cloned into pGEM-T easy vector (Promega, Madison, WI, USA) and subsequently sequenced to analyze its identity by NCBI-BLAST. In order to obtain full length *FTZ-F1*, RNA-ligase mediated RACE system (Invitrogen) was used. Primary and nested RACE primers provided in the kit and gene specific primers (5′ and 3′ RACE primers, Table 1) designed from partial fragment of *FTZ-F1* as per the manufacturer's protocol were used for performing RACE. The RACE amplicons were cloned into pGEM-T easy vector and subsequently sequenced to examine its identity by NCBI-BLAST.

### Polyclonal antibody generation and peptide affinity purification

Based on the deduced amino acid sequence of FTZ-F1, an antigenic peptide, NH_2_- KAEHPDPYSGSPDS-COOH was synthesized and conjugated to keyhole limpet hemocyanin carrier protein commercially (USV Limited, Mumbai, India). This peptide was dissolved in PBS (pH 7.4) and used for antibody production. All the rabbits used in the present study were obtained by prior permission (Approval Number: UH/IAEC/AS/BS-1-E1/2009) from IAEC and Committee for the Purpose of Control and Supervision of Experiments on Animals (CPCSEA) to generate FTZ-F1 antisera. Three-months old New Zealand white male rabbits used for antibody production were housed in our animal house facility and handled as per the animal ethical guidelines of IAEC and CPCSEA. Prior to injection, the lateral ear vein was bled to collect pre-immune serum. The animals were injected subcutaneously with 200 µg of antigenic peptide emulsified with Freund's complete adjuvant (Banglore Genie, Bengaluru, India) followed by two booster injections of antigen (100 µg) emulsified with Freund's incomplete adjuvant (Banglore Genie). The serum was collected and the presence of antibody was confirmed by a dot-blot method [45]. The antibody raised was further affinity purified using CNBr-activated Sepharose™ 4B (Amersham Biosciences, Chandler, AZ, USA) beads. In brief, the FTZ-F1 antigenic peptide was coupled to the beads. The serum was passed through sepharose column for binding and the bound antibody was eluted with 200 mM glycine (pH 2.8). The IgG fraction was checked for the presence of heavy and light chains.

### Quantitative real time PCR (qRT-PCR) analysis of *FTZ-F1*

Total RNA (5 µg) was prepared from various tissues including brain collected during different phases of reproductive cycle, ontogeny and various time points after hCG treatment. Absence of genomic DNA contamination in the total RNA was confirmed by using non-reverse transcribed samples as templates. In addition, absence of genomic DNA in total RNA was ensured by treating with DNaseI (Fermentas, GmbH, Germany) before proceeding for 1^st^ strand cDNA synthesis. The primers for qRT-PCR were designed from the conserved region of DNA binding domain of *FTZ-F1* gene (Table 1). Primers for *β-actin* (GenBank accession number JN806115), used as the reference gene, are listed in the Table 1. Primer specificity for each primer pair was confirmed by cloning and sequencing the amplicons followed by dissociation curve analysis. Real-time PCR was performed on an ABI Prism® 7500 fast thermal cycler (Applied Biosystems, Foster, CA, USA) using SYBR® Green 1 (Applied Biosystems). Each sample was run in triplicate in a final volume of 25 µl containing 0.3 µl of 1^st^ strand cDNA template, 10 pmol of each primer, and 12.5 µl of Power SYBR® Green PCR master mix. During PCR, fluorescence accumulation resulting from DNA amplification was analyzed using the sequence detector software (Applied Biosystems). Both *FTZ-F1* and *β-actin* were amplified in separate reactions using the same pool of 1^st^ strand cDNA template from each sample. Cycle threshold (C_T_) values were recorded during exponential phase of PCR amplification, the expression of *FTZ-F1* was normalized to that of *β-actin* (ΔC_T_ = *FTZ-F1* C_T_ - *β-actin* C_T_) and abundance of *FTZ-F1* mRNA was calculated using the formula 2^−ΔCT^. Tissue distribution of *FTZ-F1* and its expression in brain during gonadal ontogeny was reported as 2^−ΔCT^ whereas the expression of brain *FTZ-F1* during ovarian cycle and after hCG treatments was reported as abundance relative to the values obtained for preparatory phase of ovarian cycle and 0 h after hCG induction, *in vivo*, respectively as per the formula 2^−ΔΔCT^ and method of Livak and Schmittgen [46]. RQ Manager 1.2 (Applied Biosystems) was used to compile data from all plates and compare expression levels.

### hCG induction, *in vivo*

Different batches of female fish (n = 5) collected during preparatory (March) and prespawning (May) phases were used for hCG induction, *in vivo* experiments. Catfish were injected with 1000 IU of hCG/Kg body weight (Pubergen, Uni-Sankyo Pvt. Ltd., India), intraperitoneally as per our previous report [41]. Brain was collected at 0, 4, 8, 12 and 24 h time points. Controls were treated with physiological saline (vehicle).

### SDS-PAGE and Western blot analysis

Different tissues including brain from adult prespawning phase catfish in addition to the brain of preparatory phase catfish were collected and snap frozen in liquid N_2_ and homogenized in protein extraction buffer [10 mM Tris-Cl (pH 7.4), 0.1% Triton X-100, 1 mM PMSF, 1 mM EDTA and 1 mM DTT] followed by centrifugation at 500 x g for 15 min to remove debris. To avoid the loss of protein activity of samples, the aliquots of the supernatant were then used immediately to perform tris-glycine sodium dodecyl sulfate-polyacrylamide gel electrophoresis (SDS-PAGE, 12%) with acrylamide:N,N'-bisacrylamide (30∶1) according to the procedure of Laemmli [47]. In brief, the sample was prepared by mixing 100 µg of protein homegenate with the sample buffer containing 0.125 M Tris-Cl (pH 6.8), 4% SDS, 20% glycerol, 10% 2-mercaptoethanol and 0.002% bromophenol blue followed by incubation at 100°C for 1 min. Tris-glycine [25 mM Tris and 192 mM glycine (pH 8.3)] with 0.1% SDS was used as the electrode buffer and electrophoresis was carried out at 100 V.

Gel segregated polypeptides were transferred (electro-blotted) onto nitrocellulose membrane using Trans-Blot apparatus (Bio-Rad, Hercules, CA, USA) according to the method of Towbin *et al*. [48] **.** For this, the gel was first equilibrated in Towbin buffer (25 mM Tris, 192 mM glycine and 20% methanol) for 30 min followed by transfer to the membrane for 3 h at 70 V with 250 mA current limit. For immunostaining, the protein blot was treated with 3% BSA (w/v) in tris-buffered saline (TBS) [10 mM Tris-Cl (pH 7.4), 150 mM NaCl and 0.1%) for 1 h at room temperature to block non-specific binding followed by washing with TBST (TBS containing Tween-20 (v/v). The blot was then incubated with the FTZ-F1 (primary) antibody (1∶500 dilution in TBST containing 3% BSA (w/v)) for 2 h to overnight. After thorough washes in TBST, the blot was incubated with ALP (alkaline phosphatase) conjugated anti-mouse or anti-rabbit IgG (Banglore Genie) for 1 h and then washed again with TBST. The visualization of the specific immunoreactivity was tested using the substrates of ALP i.e., NBT/BCIP (0.0033% nitroblue tetrazolium and 0.0165% 5-bromo-4-chloro-3-indoly-l-phosphate; Millipore, Billerica, MA, USA) for color reaction.

### Statistical analysis

All the data were expressed as mean ± SEM (Standard Error of Mean). Significance among groups was tested by ANOVA followed by Student-Newman-Keuls' posthoc test using Sigmastat (version 11) software. Differences among groups were considered significant at *P*<0.05.
